# Undetected permanent dental inflammation as a possible trigger for brain abscesses? A retrospective analysis over the last 2 decades

**DOI:** 10.1007/s00701-024-06208-6

**Published:** 2024-07-31

**Authors:** Maximilian Olivier, Luisa Mona Kraus, Leonard Simon Brandenburg, Lukas Andereggen, Christian Fung, Jürgen Beck, Oliver Schnell, Debora Cipriani

**Affiliations:** 1 Department of Neurosurgery, Medical Center-University of Freiburg, Freiburg, Germany; 2 Department of Neurosurgery, Medical Center-University of Erlangen-Nürnberg, Erlangen, Germany; 3 Department of Oral and Maxillofacial Surgery, Medical Center-University of Freiburg, Freiburg, Germany; 4 Department of Neurosurgery, Kantonsspital Aarau, Aarau, Switzerland; 5 Faculty of Medicine, University of Bern, Bern, Switzerland

**Keywords:** Brain abscess, Oral infection, Odontogenic, Streptococci

## Abstract

**Background:**

Recently, there is increasing evidence that the proportion of odontogenic brain abscesses is greater than previously known. In this study, we aim to differentiate the oral infections as triggers more precisely and to classify them in the clinical setting.

**Methods:**

For analysis, we conducted a retrospective single center study. We reviewed patients with brain abscesses who have undergone treatment in the University Hospital of Freiburg, Germany in the period between 2000–2021. Inclusion required two main criteria: 1. The brain abscess must not have an other focus than odontogenic. 2. The microbial spectrum identified in the brain abscess must be consistent with an odontogenic origin.

**Results:**

Of 217 brain abscess patients, 26 met the inclusion criteria. 42% (11 patients) suffered from immunosuppressive conditions. Odontogenic foci were diagnosed in 18 cases (69%). Neurologic deficits included vigilance reduction and hemiparesis. Pathogens of the Streptococcus anginosus group were the most frequent causative agent (21 cases, 81%). Metronidazole (54%) and ceftriaxone (42%) were part of the targeted antibiotic therapy. All brain abscesses were surgically treated. Teeth were extracted in 14 of 17 cases for focus control. 18 cases (72%) showed complete or partial resolution of neurologic symptoms and 3 cases were fatal.

**Conclusion:**

Apparently silent or chronic oral infections are sufficient to cause bacterial colonization of the brain, especially in immunocompromised patients. Therefore, special care should be taken to maintain good oral health. An interdisciplinary management should become a standard to prevent and treat the occurrence of brain abscesses.

## Introduction

Brain abscesses are rare but dangerous diseases with an incidence of 0.4–0.8/100,000 and a mortality rate of up to 20% despite medical progress [4, 6].

Causes include neurosurgical operations or head trauma, infections in the lungs or in the head and neck region, up to endocarditis [1, 13, 17]. Intraoral foci of infection should be considered likewise [1]. Oral infections often originate from the teeth or periodontium [10]. In the past, however, in a large number of cases with brain abscesses the specific etiology remained unclear (30%) [15].

Recent evidence shows that the proportion of odontogenically caused brain abscesses is a larger proportion than previously known, accounting for a large fraction of cryptogenic infections [4, 10]. Reasons for this assumption are the bacteria found in brain abscesses, which are often germs from the normal oral flora or can be regularly isolated in odontogenic infections. Nevertheless, the specific disease that leads to cerebral spread is unclear [8].

In our study, we try to find further clues to the odontogenic triggers in brain abscesses. In doing so, we also consider the clinical course and the therapeutic interventions.

## Methods and materials

We conducted a retrospective, single-center study at the University Hospital Freiburg, Germany, from 2000–2021. All cases with a diagnosis of brain abscess coded according to the International Classification of Diseases (ICD-10) were considered. Exclusion criteria were postoperative or posttraumatic infections. Similarly, cases with insufficient clinical data or underaged patients were not included. The two main conditions for inclusion in the study were: 1. the brain abscess must have no other focus than odontogenic; 2. the spectrum of germs found in the brain abscess must fit an odontogenic origin. The use of all patient-related information was in accordance with the ethical standards of the Institutional and National Research Committee and the Declaration of Helsinki and its later amendments. We obtained approval from the local ethics committee Freiburg (22–1253-S1).

The parameters collected from the patient records can be seen in detail in Table 1. The focus of the data collection was on an oral etiology and previous dental treatment. We also collected information on demographic data, possible risk factors, radiological imaging, diagnoses and treatment as well as clinical parameters such as symptoms, neurological diagnostics and microbiological results.

**Table 1:** Patient characteristics and collected data

Demographics	Age
	Gender
Risk factors	Immunosuppression
	Habits
Oral health	Prior dental procedure
	OMS consultation
	Dental imaging
	Oral pathology
	Site of oral pathology
	Treatment OMS
Symptoms	Presenting signs and symptoms
	Temperature
	Neurologic evaluation
	Outcome
Laboratory values	Leukocytes
	C-reactive protein
Neurological imaging	CT
	MRI
	MRI ADC + DWI
	Site of brain abscess
Microbiology	Microbiology brain abscess
	Blood cultures
	CSF cultures
	Microbiology oral pathology
Therapy	Antimicrobial therapy
	Treatment Neurosurgery
	Recurrence
	Specialties involved

*OMS* oral and maxillofacial surgeon, *CT* computed tomography, *MRI* magnetic resonance imaging, *ADC* apparent diffusion coefficient, *DWI* diffusion-weighted imaging, *CSF* cerebrospinal fluid

Statistical analysis of the data was performed using SPSS Statistics, Version 22.0 (IBM Corp, Armonk, NY, USA). Mean, frequency, range and standard deviation (SD) were calculated as far as allowed for each variable. To examine the association between variables, the chi-square test with a significance level of 0.05 was applied.

## Results

### Clinical characteristics

**Fig. 1:**
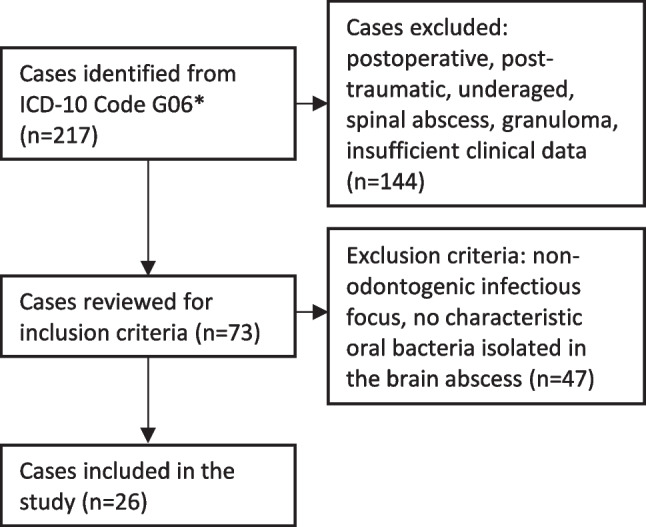
Identification of cases for inclusion. *Intracranial and intraspinal abscesses and granulomas

After application of inclusion and exclusion criteria, we eventually investigated 26 cases in our study (Fig. 1). Of the 26 patients with brain abscess, 12 were female and 14 were male. The age range was from 31 to 81 years and the mean age was 59 years. 58% (15 patients) of the patients were not suffering from immunosuppressive conditions. In contrast, 42% (11 patients) had a history of one or more risk factors (malignancies 27%, autoimmune diseases 45%, diabetes mellitus 36%, immunosuppressive drugs 18%). The use of addictive substances was present in 6 patients (23%). Among these, smoking was the largest proportion (67%). The most common symptom with which the patients presented to the emergency department was headache (14 patients; 54%). The other symptoms were: Vomiting (23%), seizures (23%), decreased level of consciousness (19%), hemiparesis (19%), nausea (12%), aphasia (12%), hemihypesthesia (12%), dizziness (8%), disorientation (8%), meningism (4%), visual impairment (4%), dysarthria (4%).

### Oral health

Just before the development of the brain abscess, 5 patients (19%) visited the dentist for the following reasons: Extraction (80%), abscess incision (20%) and not reported (N.R.) (20%). Prior to 2010, an oral and maxillofacial surgeon (OMS) was consulted in only one in five cases. Consequently, there was also no evidence of an oral focus in the other 4 cases. After 2010, however, OMSs were involved in all cases (21 patients).

**Fig. 2:**
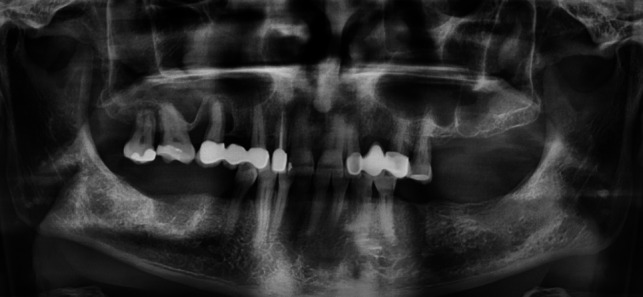
Panoramic radiograph before treatment by the OMS. The horizontal bone accretion is consistent with periodontitis. In addition, tooth 17 and 15 (according to FDI-scheme) show apical/periradicular radiolucency consistent with apical/periradicular periodontitis. Related to this is the radiodense basal right maxillary sinus as an expression of odontogenic sinusitis

**Fig. 3:**
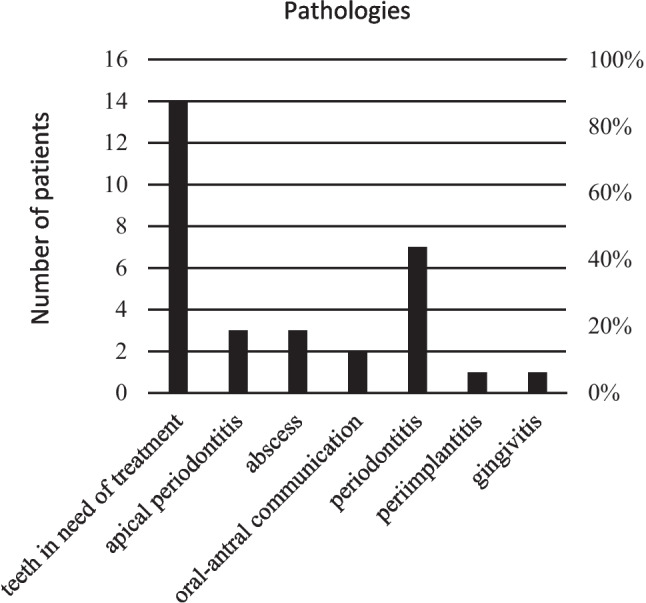
Frequency of oral cavity pathologies

Oral pathologies were found in 18 cases (69%) and 11 patients (42%) even had multiple diagnoses. Interestingly, 78% (14 cases) of patients with dentoalveolar disease had teeth in need of treatment. We define the latter in our study as a category of inadequate oral health consisting of teeth not worthy of preservation (broken teeth, root residues, deeply destroyed teeth), caries and insufficient fillings (Fig. 2). In 6 of these cases, periodontitis was also diagnosed, which was the second most common oral pathology overall (7 patients, 39%). The other diagnoses are shown in Fig. 3.

Radiological imaging was used to establish the oral diagnosis in 12 of 22 consults (55%). Nine patients (75%) underwent a panoramic radiograph, three patients (25%) underwent a computed tomography (CT) of the midface and one patient (8%) underwent a dental film or a digital volume tomography (DVT), respectively.

In terms of localization, there was a cluster in the maxillary posterior region (13 patients, 81%). In 8 cases, several sites were affected simultaneously, resulting in the following distribution: Mandibular posterior (8 patients, 50%), maxillary anterior (38%), mandibular anterior (19%). A correlation between the locations of the odontogenic focus and the brain abscess could not be identified (*p* = 0.7).

### Neurological diagnostics

In the clinical neurologic examination vigilance reduction (11 patients, 44%) and hemiparesis (10 patients, 40%) were the most common symptoms. Seventeen patients (65%) showed a combination of symptoms. The other symptoms were as follows: Aphasia (28%), meningism (20%), visual impairment (20%), hemihypesthesia (20%), disorientation (20%), coma (12%), ataxia (12%) and seizure (4%). There was no neurological status information to collect in one patient.

The mean measured temperature of the patients was 37.1 °C. The infection parameters ranged from low values in the normal range (leucocyte count 4.76 thousand/μl; CRP 3 mg/l) to highly elevated CRP values of up to 319 mg/l and leucocyte counts of up to 41.1 thousand/μl.

Considering imaging, 25 cases (96%) underwent CT for initial diagnosis. However, the one case without CT immediately received magnetic resonance imaging (MRI). In 23 cases (88%), an MRI was performed for further differential diagnosis of the lesion. Nowadays, the apparent diffusion coefficient (ADC) mapping and diffusion-weighted imaging (DWI) sequences are decisive for the confirmation of the suspected diagnosis of brain abscess. Thus, since 2012, all patients (14 patients) received an MRI with these sequences. Only 4 out of 9 MRIs before 2012, were performed with the ADC and DWI sequences.

The localization of brain abscesses was balanced (frontal 27% (7 patients), parietal 31%, temporal 35%, occipital 15%, cerebellar 8%).

### Microbiological findings

**Fig. 4:**
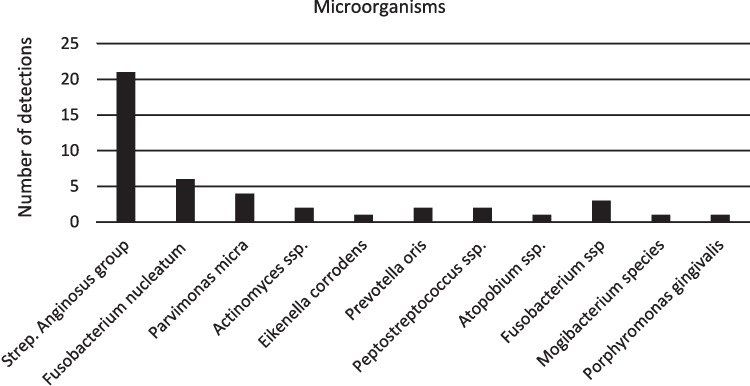
Pathogens isolated from the material of brain abscesses

Data on the microbiology of the brain abscess is available in all cases. By far the most frequently isolated pathogens were those of the Streptococcus anginosus group (21 patients, 81%). It consists of gram-positive bacterial strains with very similar characteristics. These include Streptococcus anginosus, Strep. constellatus and Strep. intermedius. Overall, mixed infections were present in 27% of cases. Fusobacterium nucleatum with 23% (6 patients) was the second most common pathogen, followed by Parvimonas micra (15%), Fusobacterium ssp. (12%), Actinomyces ssp. (8%), Prevotella oris (8%), Peptostreptococcus ssp. (8%), Eikenella corrodens (4%), Atopobium ssp. (4%), Mogibacterium species (4%) and Porphyromonas gingivalis (4%) (Fig. 4).

Blood cultures were taken from 11 patients (42%) and remained without germ detection in 10 cases (91%). In only one sample, analogous to the sample from the brain abscess, bacteria of the Strep. anginosus group could be cultured. In 9 patients (35%) cerebrospinal fluid (CSF) was also collected. 5 of these samples were positive and all could detect the causative germ already before surgery. Intraoral pathogen sampling during OMS treatment was done in only 5 cases (19%). Thereby 3 samples showed completely different germs and 2 samples could detect the same bacteria as those in the brain abscess.

The calculated antibiotic therapy consisted in 96% of a combination of different drugs. Mostly ceftriaxone (87%, 20 patients) was used together with metronidazole (70%) and vancomycin (61%). In 3 cases, the calculated antibiotic therapy could not be reproduced. After culturing the pathogens and determining resistance, a total of 17 different antibiotics were used. Metronidazole (54%) and ceftriaxone (42%) were the most used. Interestingly, vancomycin was not given at all in specific pathogen treatment. The average duration of therapy was 7.95 weeks (SD: 6.07), with the longest period being over 32 weeks.

### Surgical procedure and outcome

Of 17 patients in whom procedures were performed by OMSs, 14 (82%) involved the extraction of teeth. Thus, of the patients with teeth in need of treatment as the presumed source of infection, only 3 patients did not have teeth extracted. Other therapeutic measures for focal control were: Dental treatment (18%), periodontal therapy (18%), oral-antral communication repairment (12%) or implant removal (6%).

From the neurosurgical side, all patients underwent surgery. Of these, 18 patients (69%) underwent craniotomy with evacuation of the abscess and 5 also had a drainage. 27% of the patients received a burr hole trepanation with abscess drainage and one abscess was drained stereotactically (4%). There was no correlation between surgical technique and patient survival or recurrence (*p* = 0.4; *p* = 0,6).

After surgery, symptoms in neurological status disappeared or regressed in the majority of the cases (18 patients, 72%). In 4 patients (16%), the clinical picture was constant. Of all patients, symptoms worsened in 3 cases (12%), resulting in death during hospitalization in all 3 patients. In one case, there is no postoperative information. Recurrence developed in 5 patients (19%) during the course.

In all cases, several departments were involved in the treatment. The exact collaboration was composed of neurosurgery (26 cases, 100%), radiology (100%), institute of microbiology (100%), oral and maxillofacial surgery (85%), otorhinolaryngology (54%), neurology (27%) and internal medicine (23%).

## Discussion

Brain abscesses are often associated with a compromised immune system, as was confirmed in our study. Causes were malignant tumors, autoimmune diseases or diabetes mellitus [1]. In addition, health impairing habits such as alcohol consumption also weaken the immune system and are considered risk factors for brain abscesses [1]. Here again, 1 in 5 patients exhibited a health-damaging habit in our cohort. Similarly, odontogenic infections show a clustering in immunocompromised patients [16]. The spread of odontogenic infections is also favored by preexisting conditions such as diabetes mellitus, which may explain the high prevalence of the latter in our study [18]. Thus, both oral infection as a cause and brain abscess as a consequence are dependent on the patient’s immune response. Nevertheless, in some cases, apparently healthy patients are also affected.

Almost 20% of the patients studied visited their dentist immediately before the brain abscess developed. The most recent nationwide studies from Denmark can even establish no connection at all between visits to the dentist and brain abscesses [2]. This raises the question of whether dental treatment, as is often assumed, is the starting point for a brain abscess. On the contrary, even the avoidance of dental treatment could be part of the cause.

Only in the context of the focus search possible oral triggers are then found. A wide range from previous dental prophylactic treatments to abscess is discussed [8, 10, 11]. In our study, in addition to some acute oral processes such as abscesses, mainly chronic bacterial foci were found. These were periodontitis or decayed teeth. The basis for these diseases is often inadequate oral hygiene and irregular dental visits and it is noteworthy that the clinical course is frequently inconspicuous. This leads to permanent pathological accumulations of bacteria, which can spread throughout the body at any time [14]. Thus, there is a greater presumption that in order to prevent brain abscesses, the long-term maintenance of a healthy dental status is essential.

In our analysis, oral infections most frequently originate from the upper molars. Some studies see this as an argument for continuous spread of bacteria, especially to the frontal lobes [10, 12]. However, it should be noted that molars are the most common site of odontogenic infections overall [7]. In addition, we could not find any correlation between the teeth involved and the location of the brain abscess. Thus, a hematogenous distribution of the bacteria is more likely.

A meta-analysis has provided information that various types of streptococci, including viridans streptococci or also known as oral streptococci, are the main causative organisms in brain abscesses [5]. However, an odontogenic focus was reported as the cause in only 5% of cases, and the cause remained unclear in almost 20% of cases. A reason for this could be the unremarkable course of infections of the teeth or periodontium as described above. In our analysis, streptococci of the anginosus group, which belong to the superordinate group of viridans streptococci, were found in 81% of cases. Mixed infections were found in a quarter of the abscesses. Other studies that focused exclusively on odontogenic brain abscesses consistently showed viridans streptococci as the most common pathogens [8, 10, 16]. Gram-negative Fusobacterium ssp. also play a major role, followed by a whole plethora of other bacteria that can be the trigger in individual cases.

In our data, blood cultures showed the causative germ in less than 10% of cases. This rate is even lower than described in the literature [5]. Thus, a negative blood culture does not rule out the brain abscess despite the presumed hematogenous seeding. A possible explanation would be a transient bacterial load spike in the blood. This could be triggered analogously to bacteremia during tooth brushing by even the slightest manipulation of the infection site [14].

After determining the germs and resistance, metronidazole and ceftriaxone were among the therapies of choice in about half of the odontogenic brain abscesses. These two are also part of the national guideline on calculated antibiotic therapy [9]. However, due to the wide spectrum of oral germs and their resistances, microbiological cultivation remains essential. In addition, the average duration of antibiotic administration, 8 weeks, was twice that reported in the literature demonstrating prolonged therapy in odontogenic brain abscesses [3].

Most studies report therapy for brain abscess, but not for oral focus. Of the few reports, tooth extraction is the most common treatment, accounting for around a quarter [8, 11]. At our institution, extraction is still much more common at over 80%. Certainly, extraction represents the most thorough and rapid therapy for focal control in this diagnosis in the setting of brain abscess, but it is also the most invasive. If dental treatment needs were recognized before the brain abscess develops, tooth-preserving therapy might also be sufficient in some cases.

All patients in whom the neurological status worsened after surgery died. In all of them, significant limitations ranging from hemiparesis to coma could already be seen on admission, which seems to be a prognostic factor [19]. Overall, the lethality is 14% which is in line with previous expectations [3, 5].

Due to the rarity of brain abscesses and the monocentric study design, our study is limited by the small number of cases and the associated statistical uncertainty. Likewise, the follow-up could just be taken from the medical records to a limited extent and therefore only the immediate outcome could be considered. In addition, cases with an oral germ spectrum but no focus found at all were included in order to be able to consider the possibility of inconspicuous or undetected oral infections. As oral pathogens are not exclusively found in the oral cavity, overlooked, more distant infections could also have been the cause. A prospective approach with a detailed oral focus search and simultaneous pathogen diagnostics of brain abscess and oral focus could provide evidence in favor of our thesis.

In conclusion, we were able to show in the 26 patients that brain abscesses can be rare, multifaceted, and very dangerous consequences of odontogenic infection. Apparently inconspicuous or chronic oral infections might be sufficient to cause bacterial colonization of the brain. Patients with a weakened immune system are often affected. Thus, special care should be taken to maintain good oral health in this cohort.

## Data Availability

The datasets generated during and/or analyzed during the current study are available from the corresponding author on request.

## Abbreviations

ADC: Apparent diffusion coefficient
CRP: C-reactive protein
CSF: Cerebrospinal fluid
CT: Computed tomography
DVT: Digital volume tomography
DWI: Diffusion-weighted imaging
MRI: Magnetic resonance imaging
N.R.: Not reported
OMS: Oral and maxillofacial surgeon
SD: Standard deviation
